# Impact of flexible noise control (FNC) image processing parameters on portable chest radiography

**DOI:** 10.1002/acm2.13812

**Published:** 2022-11-02

**Authors:** Krystal M. Kirby, Liqiang Ren, Timothy R. Daly, Yasmeen K. Tandon, Brian J. Bartholmai, Beth A. Schueler, Zaiyang Long

**Affiliations:** ^1^ Department of Radiology Mayo Clinic Rochester Minnesota USA

**Keywords:** chest, digital radiography, FNC, noise reduction

## Abstract

There is a lack of understanding in the performance of flexible noise control (FNC) processing, which is used in digital radiography on a scanner vendor and has four parameters each involving multiple options. The aim of this study was to investigate the impact of FNC on portable chest imaging. An anthropomorphic chest phantom was imaged using a clinical chest program with 85 kV and five radiation dose levels at 40″ source‐to‐image distance with software‐based scatter reduction method. All images were processed without and with FNC. Noise analysis was performed in two regions of interest (ROI) on subtracted noise‐only images, and line profiles were generated through a lung‐rib interface. In addition, noise power spectra (NPS) analysis was performed in solid water phantoms of 10 and 20 cm thicknesses, using the same acquisition program and a range of dose levels. Last, feedback on retrospectively deidentified, reprocessed, and randomized clinical images from 20 portable chest exams was gathered from two thoracic radiologists. Noise reduction performances of FNC were demonstrated, with the level depending on specific FNC parameters, dose levels, ROI placement, and phantom sizes. Higher frequency textural patterns were revealed through the NPS analysis, which varied based on FNC parameters, dose levels, and phantom sizes. Overall, the vendor default parameter FGA0.5 yielded the highest noise reduction and textural artifacts. Radiologist feedback showed consistent preference of no FNC due to the presence of textural artifacts in the FNC‐processed images. An algorithm improvement to avoid introducing artifacts would be desired.

## 1 INTRODUCTION

Chest radiography typically constitutes the largest volume of exams in general radiography practices. Portable digital radiography systems are commonplace in hospital and emergency room settings where a patient's clinical condition may prevent safe transport to a fixed radiography room. Recently, the COVID‐19 pandemic also resulted in an increased utilization of portable chest exams.^1^

In our practice, Fuji FDR Go and FDR Go PLUS models (Fujifilm Healthcare, Lexington, MA) are utilized for portable chest radiographs. There were clinical complaints regarding textural artifacts in adult chest images. Through initial investigation, these exams were found to employ a noise reduction processing algorithm, called flexible noise control (FNC), which had likely caused the artifacts. FNC is default on for all programs, involving four adjustable parameters (FFC, FNB, FNT, and FNE) with up to ten options each to suit a specific program (Table 1). In our understanding, FFC is the filter control type depending on the radiation dose level for various protocols, FNB is the balance type that controls noise depending on the image spatial frequency components, FNT controls the relationship between FNC behavior and image density or pixel value in the vendor specific processing, and FNE controls the overall amount of FNC to be applied. The effectiveness of an older version of FNC had been evaluated in computed radiography over a decade ago.^2^ However, the proper selection of the current FNC parameters remains unclear as does the impact on image quality. Therefore, this study aimed to investigate the impact of FNC on portable chest imaging.

**TABLE 1:** List of flexible noise control (FNC) parameters, descriptions, and options selected for this study

Processing parameters	Provided options	Selected options
Filter control type of FNC (FFC)	C, F: for chests A, B, D, E, G, H, M, N: for other protocols and applications	C, F
Balance type of FNC (FNB)	A, B, C, D, E, F, G: controls noise depending on spatial frequency components	A, C, E, G
Type of FNC (FNT)	A, B, C: controls relationship between FNC and density or pixel value in vendor specific processing	A, B, C
Enhancement of FNC (FNE)	0.0–1.0: overall amount	0.1, 0.5, 0.9

## 2 METHODS

### 2.1 Chest phantom imaging

An anthropomorphic chest phantom (The Phantom Laboratory, Salem, NY) was imaged using the clinical adult anterior–posterior (AP) chest program, including the use of Virtual Grid (VG, Fujifilm Healthcare, Lexington, MA), which is a software‐based scatter reduction method.^3^ Acquisition parameters included source‐to‐image distance (SID) = 40″ and kVp = 85 kV. Five levels of exposure were 0.7, 1.0, 1.4, 1.8, and 2.2 mA s, which yielded deviation indices (DI) of −2.5, −1.1, 0.3, 1.4, and 2.2, respectively, with a previously optimized target exposure index of 625. All acquisitions were repeated twice.

Each image was subjected to additional FNC processing (Table 1) and compared with the baseline image without FNC. The specific FNC parameters were denoted by combining the appropriate letters. For example, FGA0.5 represents options F for FFC, G for FNB, A for FNT, and 0.5 for FNE.

**FIGURE 1:**
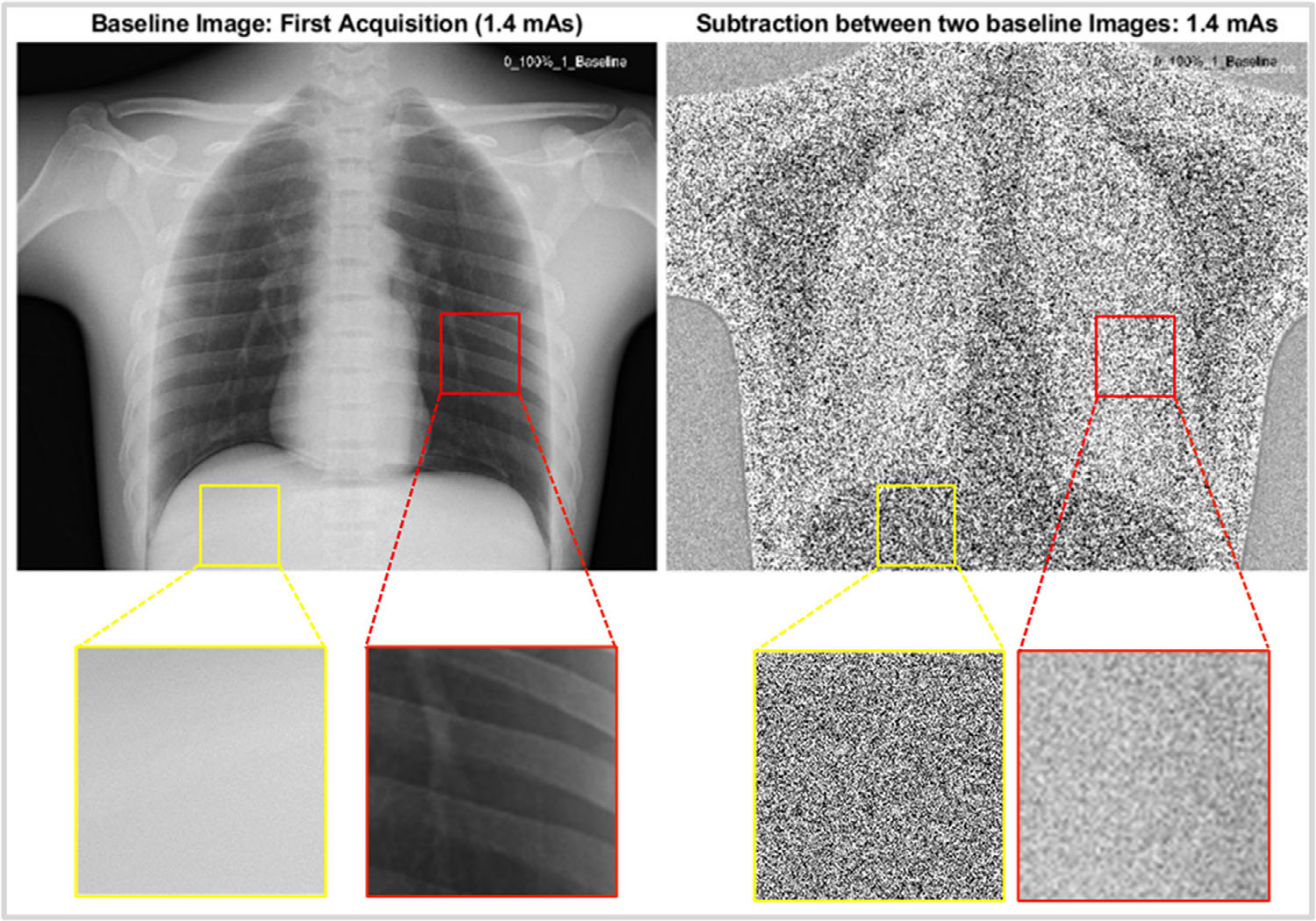
Example of original baseline image and subtraction between baseline repeats with two regions of interest (ROIs) (liver and lung), for 85 kVp and 1.4 mAs.

All analyses were performed in MATLAB 2019b (MathWorks Inc., Natick, MA). Phantom image analysis included a noise reduction analysis, in which the two repeated images were subtracted to generate a noise‐only image. Two regions of interest (ROIs) were automatically placed over the lung field and the liver area on the noise‐only image (Figure 1). Noise reduction percentage for each ROI was calculated as the difference of standard deviation between an FNC setting and baseline divided by the standard deviation from the baseline. In addition, edge preserving capability was assessed by plotting a line profile through a lung/rib interface. Importantly, if the options in a parameter were found to operate the same, that is, no pixel value difference among images processed with the various options, only one option was kept in the remaining analysis to reduce redundancy.

### 2.2 Solid water phantom imaging

To investigate the artifactual texture, uniform solid water phantoms of 10 and 20 cm thicknesses were also imaged with the clinical AP chest protocol (SID = 40″ and 85 kVp). Exposures included mAs stations of 0.32, 0.4, 0.5, 0.63, and 0.8 mAs (DI values −0.9, 0, 0.9, 1.8, and 3.1) for the 10 cm phantom. For the 20 cm phantom, mAs stations of 0.9, 1.4, 1.8, 2.2, 2.8, and 3.6 mA s (DI values of −2.8, −0.9, 0.2, 0.8, 1.5, and 2.3) were used. All acquisitions and processing were similar to the previous ones. Noise power spectrum (NPS) analysis^4^ was performed on the subtraction images from the two repeated exposures, as well as the subtraction images of the FNC‐processed and the baseline image. Specifically, ten rectangular ROIs of 128 × 128 pixels that were circularly arranged (diameter: 600 pixels) were selected and the NPS was calculated on each ROI and then averaged to determine the resultant NPS.

### 2.3 Radiologist feedback

Portable AP chest images of 20 patients were deidentified and retrospectively processed with four sets of FNC settings (FGA0.5, CGB0.5, CGC0.5, and no FNC). FGA0.5 was the vendor default setting for this program, whereas CGB0.5 and CGC0.5 were selected based on findings from the phantom studies. The images were randomly annotated with letters (A, B, C, or D) and the orders of the settings were randomized for each patient. Two fellowship‐trained thoracic radiologists, with 5 and 20 years of experience, individually reviewed the images side by side in a typical reading room setting and were tasked with identifying the best image for each patient and briefly documenting the reason. The feedback portion was based on a clinical quality improvement project.

## 3 RESULTS

### 3.1 Chest phantom imaging

Based on comparisons between FNC‐processed images and the corresponding baseline images, options for parameters FNB and FNE were found to behave the same, that is, no differences in pixel values in images processed with the various options within each parameter, over the range of mAs values. Therefore, all subsequent analyses used only the following unique FNC combinations: CGA0.5, CGB0.5, CGC0.5, FGA0.5, FGB0.5, and FBC0.5.

**FIGURE 2:**
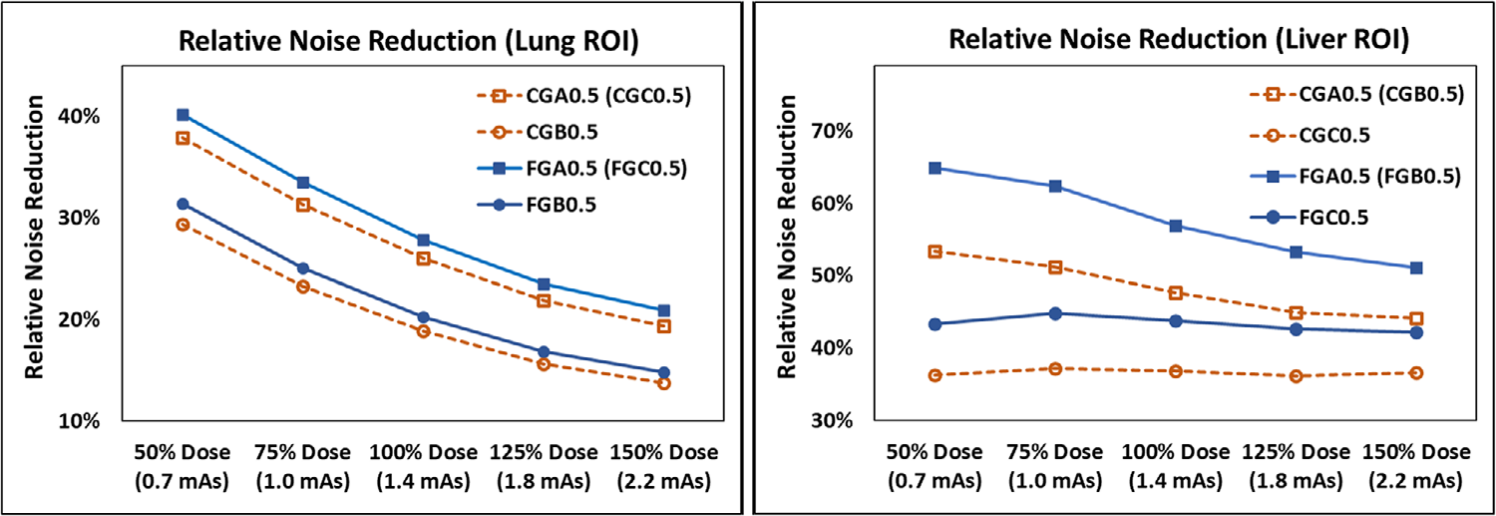
Relative noise reductions in the lung and liver region of interest (ROI) for six sets of flexible noise control (FNC) parameters. Note the identical line profiles of two FNC settings in each ROI, and the greatest noise reduction in the system default FGA0.5.

**FIGURE 3:**
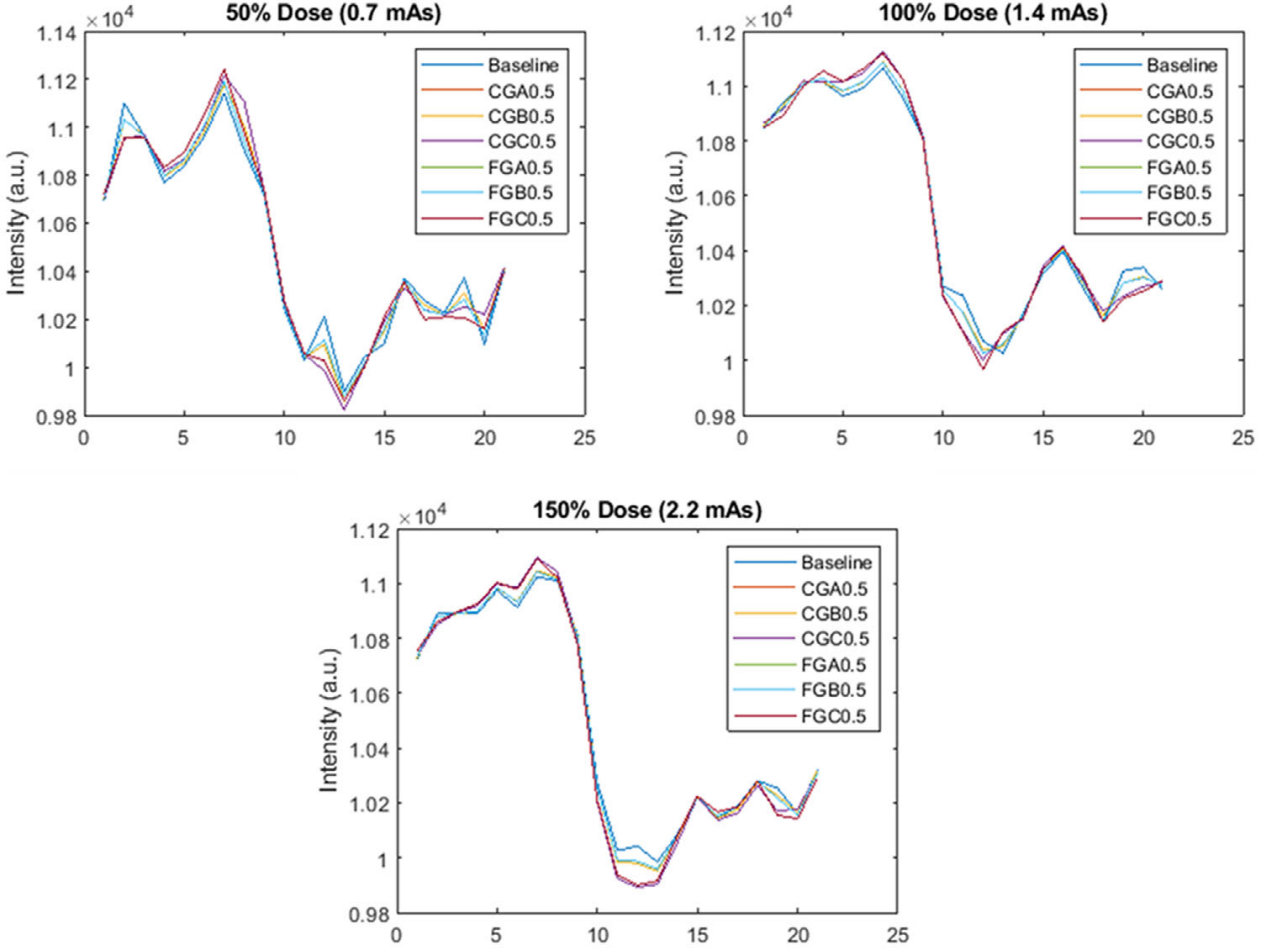
Line profiles through a lung/rib interface.

Relative noise reductions of 13.7%–64.9% were achieved with FNC based on the ROI analysis, with the exact level depending on the dose level, FNC parameter choices, and ROI selection (Figure 2). In the lung ROI, relative noise reduction increased with decreasing dose level for all FNC options, and FGA0.5 and FGC0.5 resulted in the highest noise reduction, whereas CGB0.5 was the lowest. In the liver ROI, FGA0.5, and FGB0.5 resulted in the highest noise reduction, whereas CGC0.5 was the lowest. Furthermore, line profile analysis across the FNC settings showed different edge‐preserving capabilities. CGB0.5 and FGB0.5 were closest to the baseline from three dose levels in Figure 3. Line profiles for CGA0.5 and FGA0.5 were identical to CGC0.5 and FGC0.5, respectively, and thus not seen.

### 3.2 Solid water phantom results

**FIGURE 4:**
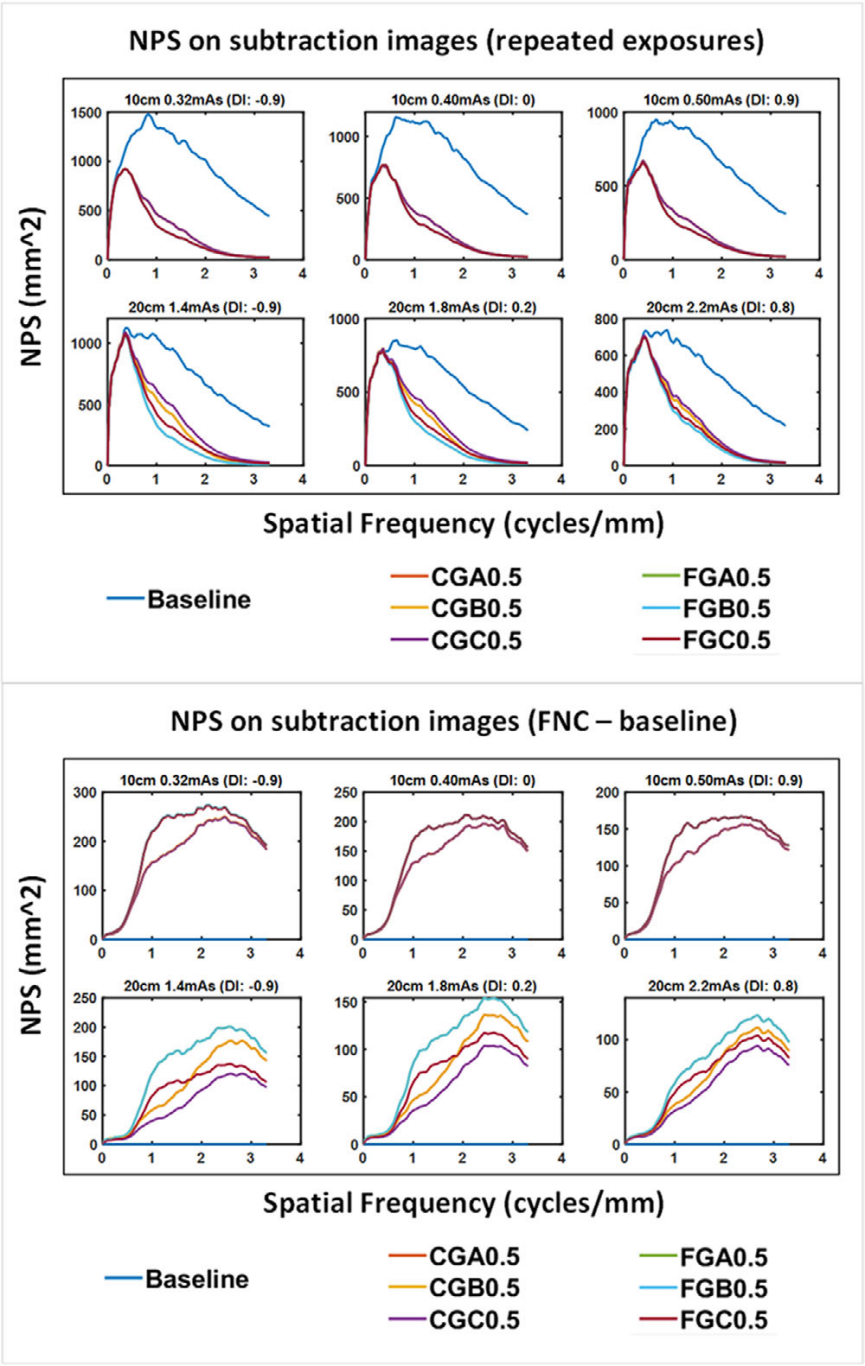
Top: noise power spectra (NPS) plotted using subtraction images acquired from two repeated exposures, for six flexible noise control (FNC) settings and the baseline image. Bottom: NPS plotted using subtraction images acquired from the FNC setting – baseline. Note: in both panels, CGA0.5/CGB0.5/CGC0.5 overlap with each other, and FGA0.5/FGB0.5/FGC0.5 overlap with each other for 10 cm phantom; CGA0.5/CGB0.5 and FGA0.5/FGB0.5 overlap with each other for 20 cm phantom.

The NPS analysis for repeated exposures is shown in Figure 4. Compared to the baseline images, the peak was shifted to lower frequencies for all FNC settings as expected. Changing the options for FNT did not show a difference for the 10 cm thick phantom. Therefore, FGA0.5 and FGB0.5 were identical to FGC0.5, whereas CGA0.5 and CGB0.5 were identical to CGC0.5. Option F in the FFC led to more noise reduction than C. In the 20 cm thick phantom, FGA0.5 and FGB0.5 overlapped, whereas CGA0.5 and CGC0.5 overlapped. Option C for FNT showed less noise reduction than A or B, particularly for the lower dose level. Moreover, NPS analysis for subtraction images acquired from the FNC – baseline image is shown in Figure 4. FGA0.5/FGB0.5 showed the highest level of high frequency patterns but the severity depended on phantom thickness and dose level. CGC0.5 consistently showed the lowest level of high frequency patterns among all FNC options.

### 3.3 Radiologist feedback

**FIGURE 5:**
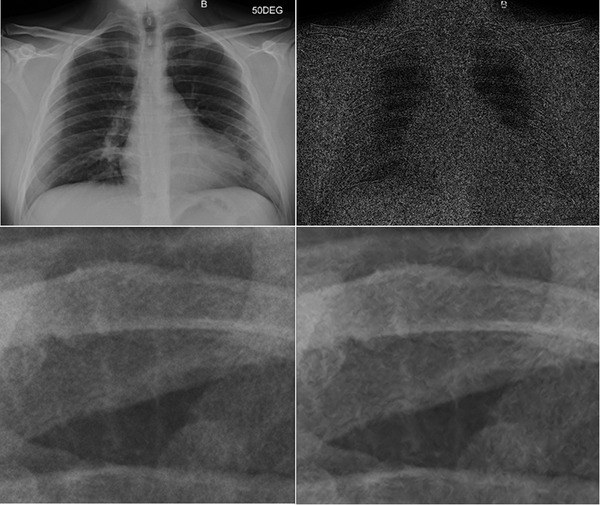
Top: (left) Image processed without flexible noise control (FNC) applied. Right: Subtraction image of the no‐FNC image subtracted from the FNC‐processed image (FGA0.5). Bottom: Enlarged view of the baseline, no‐FNC image (left), and FNC‐processed image (right), showing image noise reduction and texture artifacts due to FNC processing.

In the retrospective review of the 20 sets of images, both thoracic radiologists blindly and consistently selected the no‐FNC images as the best out of the four settings. The reason was due to the varying degrees of artifactual texture created by FNC (Figure 5). The slightly higher noise level in the no‐FNC image was not considered clinically impactful.

## 4 DISCUSSION

Quantitative assessments of image quality in chest radiography have been well studied over the last few decades^5^ using both phantoms^6^, ^7^ and clinical patient images.^8^, ^9^, ^10^ However, radiography systems often employ vendor default proprietary image processing algorithms, which could result in unexpected clinical impacts. Here, the effect of various FNC options on clinical chest imaging with software‐based scatter reduction processing (VG) was demonstrated. The reduction in noise depended on FNC parameter choices, dose levels, ROI locations, and phantom thicknesses. Noise suppressions of up to 64.9% in the liver ROI and up to 40.2% in the lung ROI were observed, which may be related to the original density differences between the two regions. The noise reduction percentage was generally higher at lower dose levels. The degree of textural artifacts was also shown to depend on FNC parameters, phantom sizes, and dose levels. Overall, vendor default FGA0.5 consistently showed the highest noise reduction and artifactual textures in phantom studies. Besides, FNC also impacts anatomy contrast such as some bone edge enhancement (Figure 5). Last but not least, four settings were selected in the retrospective radiologist feedback to provide a range of noise reduction and artifact presentation, including no FNC processing. The feedback revealed a consistent preference for clinical images without FNC between the two thoracic radiologists. The higher noise presentation was not considered clinically impactful using the current patient size‐based acquisition techniques, although better noise reduction algorithms that do not introduce artifacts and potentially allow dose reduction would be desired.

According to our understanding, the FFC parameter describes the extent to which FNC is applied depending on the radiation dose, and option F is granularity‐oriented, which provides higher noise suppression. FNC with this option increases with decreasing dose, as supported by our phantom data. Option A for FNT indicates that FNC application remains constant with respect to density. This results in more noise reduction compared to options B and C, which are supposed to decrease the relative FNC extent with increasing and decreasing density, respectively. Options for FNB and FNE showed no pixel value differences in this study setting.

Recently, algorithms have been developed to simulate the scatter reduction of a physical grid on non‐gridded images.^11^, ^12^, ^13^, ^14^ VG on our portable scanners requires a preset SID, grid ratio, and grid line rate, in order to estimate patient thickness together with exposure information and estimate scatter appropriately. Theoretically, VG and FNC behavior should be independent; however, we later discovered that FNC parameter and performance can be constrained by the existence of VG due to the inherent system design. This likely explains our observation of the different FNC parameters’ impact or the lack of impact in the current study, including that some parameter options behave the same in the FNC processing in VG exams. For example, chest phantom images processed with FCA0.9 were the same as those with FGA0.5. In our preliminary phantom evaluation of a non‐VG knee exam, many more FNC parameters demonstrated image pixel impacts.

There are extensive advanced image denoising methods from conventional to deep learning approaches, many of which were evaluated for noise reduction using phantom and/or patient images.^15^, ^16^, ^17^, ^18^ As these methods become more available in the clinical radiography practice, a careful assessment should be conducted, including potential artifacts and the loss or change of the original anatomical information. Introduction of new, especially if severe, textural artifacts may inhibit clinical interpretation. The details of FNC are vendor proprietary, but part of the algorithm may work by identifying linear and point structures and smoothing along the direction of the boundaries, which might lead to these textural artifacts. Further improvement of the algorithm to allow noise reduction without introducing artifacts would be desired for chest imaging. Images of other body parts might be impacted by FNC differently, due to factors such as the inherent difference in anatomic detail, programs with or without VG, and other acquisition techniques. Therefore, the optimal setting should be evaluated for the specific clinical use case.

This study has a few limitations. First, a vendor‐specific noise reduction algorithm was studied in a specific clinical setting. However, we expect many radiography practices throughout the world might utilize systems with this noise reduction algorithm as default. Therefore, we would like to share these study results, as well as invite more investigations and optimizations on clinical radiography processing. Second, only 85 kVp was studied because it is the only kVp in this portable chest protocol clinically. Other kVp values could be investigated in the future. Third, the phantom sizes were somewhat limited. The anthropomorphic chest phantom used is representative of a small to medium sized patient, but not the larger patients in our practice. The noise reduction may become more complex with a large phantom size, but the noise reduction behavior and textural artifacts should remain similar.

## 5 CONCLUSION

This study demonstrated the impact of FNC processing on portable chest imaging with VG regarding noise suppression and textual artifacts. A better noise reduction algorithm without introducing artifacts would be desired.

## AUTHOR CONTRIBUTIONS

Krystal M. Kirby, Beth A. Schueler, and Zaiyang Long were involved in the study conceptualization and design. Liqiang Ren and Timothy R. Daly were responsible for data collection. Krystal M. Kirby, Liqiang Ren, and Zaiyang Long performed data analysis and manuscript drafting. Yasmeen K. Tandon and Brian J. Bartholmai provided clinical expertise and retrospective imaging feedback. All were involved in data interpretation and manuscript review. All authors approved the final manuscript.

## CONFLICTS OF INTEREST

No conflicts of interest.
